# Opinions Related to the Potential Application of Artificial Intelligence (AI) by the Responsible in Charge of the Administrative Management Related to the Logistics and Supply Chain of Medical Stock in Health Centers in North of Chile

**DOI:** 10.3390/ijerph20064839

**Published:** 2023-03-09

**Authors:** Tomas Gabriel Bas, Paula Astudillo, Daniel Rojo, Angel Trigo

**Affiliations:** Escuela de Ciencias Empresariales, Universidad Católica del Norte, Coquimbo 1780000, Chile

**Keywords:** artificial intelligence, industry 4.0, administrative management, logistic, supply chain, medical stock, health centers, north of Chile

## Abstract

The research evaluated the opinion of those in charge of the administrative management of the logistics and supply chain of medical and pharmaceutical stocks of health care centers in the north of Chile and a potential improvement of their operations through the use of artificial intelligence (AI). The identification of the problem arose from the empirical analysis, where serious deficiencies in the manual handling and management of the stock of medicines and hospital supplies were evidenced. This deficiency does not allow a timely response to the demand of the logistics and supply chain, causing stock ruptures in health centers. Based on this finding, we asked ourselves how AI was observed as the most efficient tool to solve this difficulty. The results were obtained through surveys of personnel in charge of hospital and pharmacy supplies. The questions focused on the level of training, seniority in positions related to the problem, knowledge of regulations, degree of innovation in the procedures used in logistics and supply chain and procurement. However, a very striking fact was related to the importance of the use of AI, where, very surprisingly, 64.7% considered that it would not help to reduce human errors generated in the areas analyzed.

## 1. Introduction

Hospitals spend significant sums of money annually on medical and surgical supplies for the proper functioning of hospitals. In 2018, the United States spent approximately twelve million US dollars on average on supplies of this type per hospital health center across the country [1]. However, and despite being a very significant budget, the same authors estimated that to date, the improvement in logistics related to supplies is not one of the priorities of these health centers. Therefore, there is no interest in optimizing it through the use of a very powerful tool such as artificial intelligence (AI), which is one of the most disruptive technologies considered for the optimization of supply chain management, both for its impact on operations management and on production planning and control [2]. In this sense, AI has been considered for some time now, being a key enabler of different logistics initiatives such as smart production [3]. In other industries, it was demonstrated that digital transformation through the use of Industry 4.0 in the different supply chains substantially reduced process costs [4]. In some of them, these costs were even reduced by at least 50%, in addition to avoiding problems of stocks and re-stocks, which, when taken to hospitals, would be equivalent not only to reducing and optimizing costs but also fundamentally to providing certainty through greater stability of input stocks, while avoiding endangering the health and lives of the patient community [1,5]. In this sense, the fourth industrial revolution begins to play a crucial role in the supply chain due to a growing need for optimization achievable through integrated control systems adaptable to consumers, with expectations of products increasingly customized to the needs of customers and the phenomenon of temporal immediacy, generating a revolution in logistics issues [6].

It should be noted then that the use of cutting-edge technologies such as big data, blockchain, drones, robotics, augmented and virtual reality, 3D printing, Internet of Things (IoT), 5G, biometrics and, of course, AI are already part of the daily business, not only in different industries, but of common people in their daily lives [2]. The pressure exerted on healthcare facilities is increasing year by year due to, among other things, the demographic increase, the aging population, the demand for quality services, and the relentless pursuit of cost reduction. One of the most critical institutions in the health system is the hospital [7]. The same authors highlighted that the hospital staff must distribute the functions covering a large number of different departments and units, some of which are very critical, so that a wide range of skills is needed to meet the demand for all logistic activities in the hospital. Therefore, the use of these technologies for the optimal management of logistics tasks within a hospital is crucial for reducing costs, increasing the quality of care, as well as meeting the needs related to the availability of supplies in a timely manner [8]. Consequently, the optimization of administrative management of the logistics and the supply chain is paramount [5] in healthcare centers through these technologies in order to avoid deficiencies that lead to stock ruptures. In this sense, it is necessary to consider not only the critical stock itself, but also the “time factor”, which refers to the time interval that a critical input takes to reach the hospital after it is requested to the supplier from the corresponding order guide. In northern Chile, orders are usually placed manually, i.e., without the support of technologies related to Industry 4.0. However, what is serious is that the ordering protocol is often triggered when the product is no longer in stock. Therefore, the time it takes to start the process from the moment the shortage is perceived, i.e., to make the call to the corresponding supplier, to ensure that there are no typing or other errors and that the supplier, in turn, has it in stock, and so that the distribution and shipment is finally made and arrives at the hospital center, can be extremely critical for the quality of care and the life of patients due to the excessive time from order to delivery.

The identification of the problem of this research arose from the empirical experience of the lack of medicinal, medical, and pharmaceutical materials experienced by hospitals in the north of Chile due to the lack of use of tools related to Industry 4.0, particularly AI and Logistics 4.0, as well as Supply Chain 4.0. The seriousness of the problem is such that it could eventually become a major health crisis in the medium and long term, without considering the economic losses that this will cause.

In this context, optimization mechanisms related to Industry 4.0, Logistics 4.0, and artificial intelligence (AI) are seen as a low-cost solution that can be quickly implemented in the hospital sector. This would make it possible to efficiently solve this serious deficiency of manual use, because AI orders are managed with virtually no human intervention, thus enabling a constant dynamic of inputs, without losses due to shortages, or obstacles of the time factor, or unnecessary expenses due to unused or duplicated surpluses [9].

The elements that make up Industry 4.0 when they integrate highly complex global information supply networks establish separate solutions, known as Logistics 4.0 [10]. The term Logistics 4.0 refers to an integral use of different types of technologies that allow increasing both the efficiency and effectiveness of the different supply chains [4] aimed at a continuous improvement of logistics processes, thus avoiding typical errors and interruptions in transport and storage processes, mainly due to the constant exchange of data between the actors that are part of the logistics system [10]. On the other hand, it should not be forgotten that a logistics system is the sum of its different interconnected components and not just an isolated component such as transportation or inventory management [11]. To these, we must add a crucial element that is specifically related to the logistics management of goods and products, such as those related to security, which make it possible to restrict the theft of goods or also protection, damage or loss, which in industrial quantities evidently cause great economic damage [7,12]. In this sense, Di Capua (2023) [13] proposed an Integrated Logistics Platform 4.0, which aimed at the interaction and cooperation of the components used for Industry 4.0, with human resources and organizational processes formed for this purpose. On the other hand, Oleśków-Szłapka et al. (2019) [10] estimated that Logistics 4.0 not only seeks to neatly replace human error but, together with AI, is considered the promoter for Industry 4.0 from the verification of large amounts of information that allows distinguishing present and potential problems and threats, and the fastest and most economical ways to solve them. In this regard, it is important to note that the term AI was coined by John McCarthy (Dartmouth College), Marvin L. Minsky (MIT), Nathaniel Rochester (IBM), and Claude Shannon (Bell Laboratories) in 1955, who defined it as the ability of a machine to present cognitive capabilities similar to those of humans, such as reasoning, learning, creativity, and the ability to plan various functions [14,15]. The availability of big data, the innovation of algorithms, and the rapid progress of different computer analysis and processing techniques allow technological systems to perceive their environment, relate to it, solve problems, and act with a specific purpose, adapting their behavior to certain circumstances and analyzing the effects of previous actions [16]. In the field of medicine, AI techniques advanced drug design, shortening research and development times as in the case of the development of vaccines against COVID-19, increasing the possibilities of cancer detection, and high-precision medical image analysis [17,18]. However, despite the specific weight of the use of AI in medicine, there is still resistance from the ethical point of view regarding its application in humans. On the other hand, it is important to emphasize that this research does not seek to design or develop AI applications, but rather to highlight the serious deficiencies in the management and logistics of hospital supplies stock carried out manually in relation to the potential efficiency that the use of AI-derived tools could have in this area. 

However, the United Kingdom, Japan, and China made financial commitments to create research programs that integrate AI systems to increase efficiency in the diagnosis of diseases and infections within hospitals. The aim was to improve the validation factor in finding and recognizing patterns based on symptoms, history, and laboratory results. In this way, it was possible to automatically identify diseases and infections, such as meningitis, with diagnoses of up to 90% accuracy analyzed by AI [19].

Another important concept within logistics is the supply chain, which can be defined as the activities related to the transformation of a good, from the raw material to the final consumer by meeting the demands of a customer [20]. Therefore, its main objective is the distribution of the necessary materials in the required quantity, quality and time at the lowest possible cost, thus delivering a better service to end users [12]. This same medium argues that until not long ago, the relationship between supply chain and logistics mechanism was considered highly complex and costly for companies, instead of being seen as a tool that allows generating competitive advantages, while providing substantial savings in all associated operating costs. On the other hand, it should not be forgotten that every supply chain requires adequate management through resources, both human and information, and processes related to the supply chain [20]. Therefore, logistics as well as the supply chain are constantly evolving so that the delivery of inputs is accurate, both in terms of the specific customer and the timely delivery. For quite some time now, not only were Industry 4.0 and Logistics 4.0 involved, but there is already talk of “Intelligent Supply Chain” or Supply Chain 4.0 from the revolutionary combination and integration of different information technologies that allowed countless benefits and improvements such as increased efficiency and responsiveness to an order and distribution of inputs [21,22]. This makes the lack of interest on the part of hospital centers in the use of technological tools such as artificial intelligence to optimize supply chains and associated services inconceivable [1]. Therefore, through a Supply Chain 4.0, obtaining a competitive advantage is sought and what Gunasekaran et al. (2017) [21] cited as the triple A: “agility”, “alignment”, and “adaptation” must be developed to support it. This includes speed of response of the supply chain to different unexpected changes in market demand. “Alignment” through the integration of processes from different components of the supply chain to achieve better performance. Finally, the “adaptation” of the supply chain, which refers to an adaptive evolution in a timely, but also cost-effective, manner following market changes in terms of strategies, products, and technologies [21]. In this regard, it is important to note that there are different types of supply chains depending on the objectives set by the organizations. For example, some provide logistics services, which generally consist of a logistics service integrator and several functional logistics service providers, which are increasingly used [23].

Hospital leaders face different challenges in the quest to improve traditional measures in the supply chain of different inputs, but one must also consider government policies and state support institutions depending on the country addressed, as well as the different barriers that may arise [20,24,25]. In this regard, it should be noted that the uninterrupted provision of hospital supplies is crucial. In addition, it is important to note that the implementation of the health care supply chain is extremely complex to manage due to medical and pharmacological specialty issues, but also because of the interaction with human health care supplies, which has ethical implications [26]. With this, it can be inferred that if the supply chain does not function as it should, it compromises the entire hospital service process, which can lead to loss of human life, economic losses, and possible lawsuits, among other serious problems.

In this dynamic of opinion related to the potential use of artificial intelligence by professionals in charge of the logistics and supply chain of medical stock in health centers in northern Chile, it is important to consider stock rupture as a high impact variable. Stock-outs often result in substantial revenue losses for all involved, as many may be tempted to change suppliers, switch to competitors, or delay the purchase over time, generating a negative impact on customer satisfaction and the coffers of the affected organization [27]. This stock out can be measured from the simple lack of the required input at the right time to its substitution by another, where equality or lack of quality, price, origin, time interval, and availability in required quantities come into play. There is little research that addresses this last variable, since studies focused mainly on consumer reactions in terms of changes in the actions of their choices [28,29]. Because of this, these authors believe that it is very critical to consider that an input may be available at the time the order is placed, either online (with the massive arrival of e-commerce) or in person; however, it may already be out of stock when the time comes to ship, and so, the original order must be replaced by another with similar characteristics or wait for the supplier to be able to access the original product again. Substitution based on stock-outs due to unavailability of a product is very critical [30]. Stock-outs are a major source of inefficiencies and uncertainty in the supply chain within health care facilities. 

There are several reasons why it is important to maintain the stock of supplies at adequate levels for the normal performance of the health center through optimal logistics and satisfactory stock management. In this same line, the following reasons can be identified for which it is essential and necessary to maintain the stock in a hospital center.

- It allows to improve the quality of service: maintaining stock allows to respond in a timely manner to emergencies and patients’ needs as efficiently as possible, even if there is an unforeseen increase in demand.
- Reduces financial and strategic costs: a reduction in operating, transportation, and distribution costs is generated, while allowing potential savings to be reinvested in areas where capital injection needs to be reinforced.

These assumptions are reinforced due to the work carried out by Guo and Wang (2023) [9], who reached similar deductions about the constitution of stocks, where he stated that these reasons were related to customer service and the cost of savings derived from their constitution.

In Chile, the quality of hospital services was a highly criticized point by society, demanding over the years less waiting times, improvements in the quality of service, better professionals, lower costs, among other demands that were fueled by the quality of public health services.

Pizarro (2020) [31], in his study for the optimization of the supply chain of the surgical wards unit of the Dipreca Hospital (Dirección de Previsión de Carabineros de Chile/hospital of the Chilean police’s medical welfare department) in Santiago Metropolitan region (Chile), showed that there were inefficient tenders for the purchase of supplies, little planning regarding storing supplies, and a deficit in the provisioning. In addition, the author raised a clear problem, which was to formulate, in a complete and consistent way, a methodology that optimized the adequate quantities of supplies to avoid stock ruptures. This research provided the methodological basis for exploring new solutions to these inefficiencies in hospital logistics in Chile. 

On the other hand, Vallejo (2021) [32] investigated the improvement of processes in the drug supply chain based on COVID-19, mentioning the way in which healthcare centers had to go beyond their capabilities, managing to optimize the available resources to respond to the healthcare needs caused by the pandemic. This study also emphasized the importance of the clinical pharmacy unit and the shortcomings of the supply process. These included staff training, drug traceability, information on actual consumption and stock, information flow, and lack of space, leading to an increase in the occurrence of errors in the planning of services and budgeting. In short, all these existing inefficiencies generated a massive dissatisfaction of the Chilean population with the country’s healthcare system.

Based on the above, the objective of this research is to determine the opinion of professionals in charge of logistics and supply chain of medical stock in health centers in northern Chile related to the potential application of artificial intelligence (AI) to improve the efficiency of logistics and hospital supply chain. This seeks to avoid stock rupture in health centers due to miscalculation of the timing of orders, errors, or omissions caused by negligence or ignorance of the intervening personnel. For this purpose, the main causes of stock ruptures in the hospital supply chain in northern Chile will be analyzed. The supply chain of health centers will be described, identifying in their internal logistics the main procedures that may use AI.

## 2. Methodology

A mixed methodology was implemented for the experimental analysis through the questionnaire technique to measure the level of efficiency of the supply chain of hospital supplies in health centers in northern Chile, through the collection and analysis of data by means of interviews and test scores. Through the interview, non-binary answers were sought, i.e., personal opinions and experiences of the people involved in the different internal logistical processes of the health centers addressed, which could not be quantified from observations at the study site. Additionally, the information collected was complemented with a documentary methodology, as it sought to validate the research on the stated objective by establishing ranges on which the responses obtained could be parameterized.

On the other hand, for the purposes of the study, reasoning by analogy was used, in which Fisher (2018) [33] made use of analogy in science as a specific study of inductive reasoning. He argued that analogy plays a substantive role in the context of discovery and draws a parallel between the topic under discussion and a situation that offers similarities to it. This is due to the lack of concrete data, as the utmost discretion and confidentiality was agreed upon in dealing with those in charge of the health centers addressed. On this basis, it was established that the centers would not provide financial data, internal results, or sensitive data that could compromise the entities and/or patients. In other words, the analysis was of an exploratory type, offering an alternative based on an explicit recognition and classified under two criteria: by its purposes and according to its means [34]. In terms of purposes, it sought to familiarize itself with a given topic on which there was little accumulated knowledge. It is characterized as an exploratory and descriptive non-probabilistic study, this being the type of analysis retained for the conduct of the present research.

Zack et al. (2019) [35] estimated that in recent years, many researchers increasingly used non-probability samples as substitutes for random samples. The reasons are numerous; for example, response rates in probability surveys decreased dramatically, which at the same time increased the likelihood of bias, while the costs of such surveys increased considerably. On the other hand, the non-probability method is characterized by a dedicated search for qualitatively representative samples, by including groups of specialists in the topic to be studied, as was the case in our research. In addition, non-probabilistic samples are low cost and can be used to improve our understanding and knowledge of the social world and in this case of the opinion of professionals in charge of logistics and Medical Stock Supply Chain in Health Centers in Northern Chile. According to Zack et al. (2019) [35] non-probability sampling should not be discarded as a valid tool to study particular phenomena with potential to generate new and valuable knowledge. This sampling method is considered less complex and easier to apply compared to its counterpart. Therefore, in this research, non-probabilistic convenience sampling was used due to the possibility of selecting the most suitable available professionals for the research considering the variable of the geographical and spatial extension of the research in the north of Chile and the variable financial costs. From this, we observed some advantages that this method had in allowing us to obtain responses more quickly and less expensively compared to probability sampling. Participants tend to be more motivated and predisposed to respond quickly compared to randomly selected individuals. However, there are also disadvantages such as possible biases that may arise, but for this, it is essential to have a sample that represents as closely as possible the population of interest, as was the case with the sample achieved in this research.

The non-probabilistic convenience sampling was carried out according to the following selection criteria:

- The respondent must belong to the staff of officials of the analyzed health center;
- The respondent’s function must belong to or be directly or indirectly related to the supply chain of medicines and supplies of the health center;
- The official must have at least one year of experience in the health center.

In consideration of the above, the size of the sample was defined by the number of people who met the previously established selection criteria; therefore, no specific sampling formula was be used for this study.

It was necessary to take into account that in the supply areas of the health center and the clinical pharmacy, both belonging to the medical supply chain and according to the previously established criteria; it was determined that the hospital centers analyzed were a total of four (the most important by structural dimension and by patients attended in four cities in the north of Chile: Arica, Antofagasta, Iquique, and Coquimbo (see Appendix A for map of Chile). The total sample size was 17 individuals who agreed to participate in the experiment, at a rate of between four or five individuals per hospital center, which was the average of those in charge directly of the supply chain of the hospital medical centers.

On the other hand, the following data collection techniques were used, which were then merged into a single instrument for the purposes and convenience of the study.

- Interview technique: The type of interview used for data collection was semi-structured, since this allowed for expanding the inquiry and asking questions that were not initially contemplated. A personal interview was conducted with the in charge of the supply and pharmacy areas (17 individuals), in which mention was made of the description of the process carried out in their respective areas, how the areas and personnel interact with each other, and the degrees of responsibility that are handled. In addition to the above, we sought to know if they had experience with stock ruptures and how efficient the computer programs provided by the public health center were in terms of the activities they performed.
- Questionnaire technique: The second technique selected for this study was the questionnaire. This tool was the survey modality that was carried out in written form by means of an instrument or paper format containing a series of questions. It is also called a self-administered questionnaire, because it must be filled out by the respondent, without the intervention of the interviewer. For the purposes of this study, the type of questionnaire used was a closed-ended questionnaire. This technique consisted of previously establishing the response options that the respondent can choose from:
  - Dichotomous, i.e., only two response options are offered;
  - Simple selection, several options are offered, but only one is chosen.

It is necessary to clarify that, in mutual agreement with the health centers participating in the experiment, the confidentiality of the workers surveyed was requested, so the instrument did not mention their names or positions, in order to avoid individualizing them, However, due to the need to record the answers given by the personnel, it was requested that the survey be answered with the support of the institutional mail of the health center, or the mail that was linked to the company in the case of not having one, in order to ensure and identify the respondent within the list of workers, even with this requirement, the identities were kept anonymous given the nature of the agreement. 

### Instrument

The instrument (Appendix B) combined the techniques described in the previous section, corresponding to the interview technique and the questionnaire technique, which largely covered questions related to knowledge about the supply and stock out of medicines, which was validated by the heads of the areas to be interviewed in the health centers.

The application of the instrument was carried out during the second semester of the year 2022, with a target population of employees directly and indirectly related to the drug supply chain, including workers with knowledge of drug supply, stock-outs, replenishment, information flow, internal policies, and information chain belonging to the areas of logistics, nursing technicians, pharmacists, and other medical staff involved in the logistics and stock of medical and pharmaceutical supplies.

The instrument had a total of 24 questions, which were divided into three initial questions that validated the criteria previously established for the sampling. Then, there were seven questions on knowledge of inventory, storage, processes, and replenishment procedures; five questions on knowledge of the area, its regulations, and the distribution process; three questions on training levels, internal policies, use and management of inventories, and storage means; and finally, three questions regarding the impact of AI.

## 3. Analysis of Results

The results correspond to the data collected through the survey conducted among hospital supply and pharmacy staff of the four hospitals in northern Chile analyzed. It should be remembered that the objective of this research was to describe different steps related to logistics and the supply chain of medical stocks in public health centers in northern Chile analyzed through the opinion of the professionals in charge related to the potential application of artificial intelligence (AI), in order to improve the efficiency of logistics and the supply chain of the hospitals themselves. As contextualization, the supply chain of the health centers studied is mainly composed of the supply area and the clinical pharmacy area. The supply area receives medicines and supplies, investigates their condition, stores them, and supplies them to other areas, as well as purchases and replenishes them. On the other hand, the clinical pharmacy area is in charge of distributing supplies to the clinical and hospital areas, being the connection point between the supply area and the nursing section.

In order to complement the survey of officials in the supply and clinical pharmacy areas, face-to-face meetings were held with the area chiefs of these sectors to define more precisely the supply chain of the health centers.

According to the interviews conducted with the supply sections, the chain started with the request for the required supplies, but there was no AI or AI-based software within the supply chain. In contrast, programs such as Excel or Masterkey (MK) [36,37], which do not use an AI to perform their functions, were used. In addition, they relied entirely on workers to handle, enter, and communicate information, and so, human error was very present, and affected the entire use of such programs. These requests were made according to requests from other areas or according to the system, which had records of missing materials. These requests were entered into an Excel spreadsheet manually typed by the operator and then added to the system managed at the health center. 

Once the order was placed, a three- to five-day wait was made for the arrival of the supplies. Then, the reception and storage team verified the quantities, the dates of creation and expiration, and the state in which they were found, and then, they were stored. Once the above process was completed, a report was made on the materials received according to the supplier, which was verified by the reception and storage manager and delivered to the supply manager to complete the process of purchasing supplies in the health center’s system. Once the drugs and/or hospital supplies were stored, the areas were notified, in the case of a specific requested material, so that they could collect the requested materials.

On the other hand, the clinical pharmacy area made requests to the supply area, which, once approved, authorized the area officials to enter and withdraw the requested supplies, following the required procedures and protocols. Finally, the clinical pharmacy area was responsible for distributing the materials and supplies within the clinic area to the nurses and physicians to treat patients.

Within the supply chain, traditional models that were highly dependent on human intervention were those that predominate in the hospital sector today. However, it was observed that their efficiency was reduced, and their planning capacity was affected by the decrease in the foresight of the future. Current training programs did not achieve the expected objectives or, on the other hand, were not carried out in sufficient quantities. All this lead to a decrease in the quality of the service delivered, control failures due to information flow failures, errors in compliance with standards and protocols. This generated stock ruptures, which were inevitable with the current models, because they were inherent ruptures due to human intervention failures. 

On the other hand, a key milestone was to determine the level of education of the respondents, which made it possible to identify the level of understanding of the different processes in which they were involved in the storage and distribution of hospital supply, in addition to other relevant topics, such as standards and protocols related to the logistics chain and the supply chain. This could provide clues to identify the reasons for stock ruptures or errors.

Regarding the level of education, 64.7% of those surveyed had a higher technical level, followed by only 17.6% of those involved with completed university studies, and the rest were equally divided between personnel with completed secondary education, secondary technical education, and incomplete university studies, with 5.9% for each category.

Currently, within Chilean society, there is great segregation with respect to access to quality university education and schools. This segregation was reflected in the results obtained by the instrument, where only a quarter (¼) of the sample was studying or had completed a university degree. This was not negligible, since motivation, competitiveness, and rewards within an organization have a high impact on the production or efficiency of services, and so, greater specialization and more studies generate better results, both for the organization and for the human resources themselves, which are perceived as more relevant and valued the greater the recognition received. In other countries, such as the United States, Japan or China, those in charge of activities related to the supply chain in hospitals were either physicians or highly trained human resources.

On the other hand, relevant aspects stood out, such as the degree of misinformation regarding inventory control, perhaps due to the above, where, according to the data collected, of the 52.9% of the population that stated that they knew the inventory management processes, only 14.2% said that they knew them in detail and in depth; so, it was understood that the flow of information, both horizontal and vertical, was highly deficient. On the other hand, there was a high level of lack of knowledge about the flow of information in the supply chain, which reached 62.6%, which had repercussions in different areas, such as pharmacy, medical center, accounting, clinic, among others. All of the above provided critical information that allowed us to deduce a strong obstruction in the distribution and procurement of supplies.

In reference to the use or knowledge about the importance of artificial intelligence in the supply chain, there was a flagrant lack of knowledge on this matter, since 71% expressed not knowing enough or not having any comprehension about the use of this type of technology. Furthermore, this lack of knowledge was also represented in possible aspects of system improvement, where the option of including an AI-based system was not an alternative that the personnel highlighted as important, given that 35.3% did not even know about these processes. However, what is most worrying is that 65% considered that AI would not help to reduce the errors generated by people in the areas analyzed, which clearly highlights the fact that there was a degree of ignorance and resistance to change, lack of updating systems, and failures in the planning of the different processes.

When mentioning the reasons for stock ruptures, the personnel identified, out of a total of 11 options given, aspects related to human capital, logistics, inputs, and implements. Thus, aspects such as data errors (70.6%), deterioration of products for different reasons (58.8%), limited space in the different warehouses (64.7%), and non-compliance of suppliers (47.1%) stood out. In this sense, three of the four reasons most selected by the participants in the surveys were due to internal shortcomings in the logistics processes, these being negative concepts related to internal logistics problems and human error.

Other interesting results were related to the relationships between the personnel involved in the supply chains, where 82.4% said they knew the people who work in the supply area. Some 65% were familiar with the rules and procedures of the area, while 60% were unaware of the parameters related to innovation and updating of methods related to the hospital supply chain. On the other hand, 82.4% said they were aware of the purchases made in the supply centers and 76.5% were familiar with the procedures applied to the reception of products. However, 50% were not familiar with replenishment activities. Fifty-three percent were familiar with inventory management techniques and 41.2% were familiar with the procedures for periodic monitoring of the supply chain. Thirty-seven% said they were familiar with the supply chain, but did not know about possible training on the subject. 

### Implications of the Study in Practical Terms

In order to reinforce the findings of the study, this section highlighted the most important implications of the research:

- Despite the very significant budgetary investments that are routinely made in the supply chains of hospitals in industrialized countries around the world in order to improve their efficiency, there was no particular interest from the responsible of supply chain logistics to focus on the use of cutting-edge Industry 4.0 technologies to improve the supply chain management of healthcare facilities. Stock control management is historically carried out manually and almost obsolete, without the proper use of tools such as AI. It was shown in the literature that such tools allow, by analogy, not only greater efficiency, but also savings of up to 50% in operational management costs, with an increase in the quality of patient care, as well as the satisfaction of needs related to the availability of quality supplies in time and form of the processes involved, as well as the positive impact on the planning and control of distribution and storage.
- From this perspective and considering health centers in northern Chile, two implications emerged from this research. On the one hand, the total ineffectiveness of the manual use system with spreadsheets currently used in Chilean hospitals that lack the use of technology and depend on the experience of those in charge of managing the distribution chain of hospital supplies and, on the other hand, the ignorance of both authorities and those in charge about the cost and efficiency benefits of adopting AI to optimize the management of the distribution chain.
- Part of the problem seemed to be that hospitals in Chile are public, with a significant bureaucracy that leads them to be inefficient in the administrative management of medical supplies logistics, preventing, in many cases, a reflection on the stocks considered critical, which means that no decisions are made or followed by actions regarding this problem. At the same time, there is no investment in hiring ad hoc professionals or developing public policies that would lead to an improvement in the quality of this administrative management, which could often mean the life or death of a patient. The research demonstrated from this fact that technological tools such as AI are of fundamental importance in increasing efficiency and reducing costs and risks, as they are proven and largely autonomous technologies, while at the same time obviating human error, which is the most common and, therefore, the most critical factor detected.
- A major implication was that since there is a shortage of professionals in charge of managing the logistics of the stock of supplies, many times a professional hired to perform nursing tasks, for example, and whose professional training is far from an administrative management activity, must assume both responsibilities at the same time. This reduces the attention given to one or the other activity. That is to say that the nurse, instead of carrying out nursing work, is carrying out stock management work, for which she lacks the appropriate skills and neglects the activities for which does have professional skills and for which was initially hired. This increases the risk both in patient care due to the absence and fatigue of the nurses and, at the same time, in the administrative management of stocks and logistics of supplies due to omissions in their handling, generating deficiencies and errors that could be catastrophic for the health of the patients involved due to the unavailability of critical supplies.
- Another important implication detected was that the implementation of an AI system for the control of medicine stock management is a low-cost solution that can be implemented quickly in the hospital sector, enabling this serious deficiency in the manual use of logistics to be resolved efficiently and quickly, since with AI, orders are managed with practically no human intervention, thus enabling a constant dynamic of supplies, without losses due to shortages or time-related obstacles. Unnecessary costs due to unused or duplicated surpluses and storage space, many of which have special needs such as refrigeration, are avoided. Therefore, controlling the variable stock of supplies becomes the most administratively important activity in any hospital center.
- From the academic point of view, a fundamental implication is to increase research involving more countries and hospital services in order to raise awareness of the importance of using AI in the administrative management of the logistics of hospital stock in everything described above.
- The study identified a finding that strengthened the study. Normally, hospital supply orders were placed manually based on the request for the required supplies, but within the supply chain, there was no AI or software based on any Engineering 4.0 logarithm. In contrast, basic programs such as Excel or Masterkey (MK) were used, which did not employ any AI to perform their functions. However, what aggravated the situation due to lack of foresight was that, in many cases, the ordering protocol was triggered when the product was no longer in stock, i.e., when it was already too late. Therefore, the time it took to start the process from the time the shortage was noticed, to the time it took to call the corresponding supplier and for the product to arrive at its destination, could have fatal consequences for a patient. This stock out can have numerous direct and indirect consequences. From the simple lack of the required input at the right time to its substitution by another, in which the quality of the input is equal or not, the difference in price, the origin of the input, the longer time interval, as well as the availability of the required quantities at the right time, all come into play. However, from the bibliographic research, it was found that there was little research that addressed this last variable.
- Within the supply chain, traditional models with a high dependence on human intervention are predominant in the hospital sector today. All this leads to a decrease in the quality of the service delivered, control failures due to information flow failures, errors in compliance with standards and protocols. This generates stock ruptures, which are unavoidable with the current models, because they are inherent ruptures due to human intervention failures. On the other hand, a key milestone was to determine the level of education of the respondents, which made it possible to identify the level of understanding of the different processes in which they were involved in the storage and distribution of hospital supply, as well as other relevant topics, such as standards and protocols related to the logistics chain and the supply chain. This could provide clues to identify the reasons for stock-outs or errors.
- An extremely worrying incidence arose from 64.7% of respondents who believed that AI would not help to reduce human errors generated by the handling of supply chain information. This is extremely serious due to the very high degree of ignorance and resistance to change on the part of those involved in hospital logistics, the lack of updating of systems and failures in the planning of the different processes, and the recruitment of suitable professional staff to handle everything related to Industry 4.0, Logistics 4.0, and, obviously, AI.

Finally, and in summary, the following can be established:

- To increase the relevance of implementing AI in the hospital management sector in the north of Chile in order to improve the logistical and operational efficiency of the supply chain of inputs by enhancing improvements in IT systems through the tools provided by Engineering 4.0;
- Highlight the most common shortcomings related to the supply chain management areas of hospitals in northern Chile;
- Distinguish the inclusion of AI to support the resolution of common problems and improve the efficiency of administrative management processes in hospitals;
- To establish an academic precedent in research on the importance of studying the optimization of hospital administrative management logistics processes based on computer systems and AI, in which there is an important gap and which is of paramount importance to increase the efficiency of hospital budgets and the security in the availability of necessary medical supplies.

## 4. Conclusions

The inefficiencies that were detected within the logistics and hospital supply chain, through the stock ruptures that these generate, are a concern at the institutional level and their potential elimination is key to the existence of a quality service. In addition, there is a need to maintain competent and updated systems that complement the achievement of the goals established by the hospital centers. This is why the approach of the present study and the fulfillment of the objectives is a possible solution, together with an improvement that could break the paradigm of the supply chain operation that currently exists in the sector, where it seeks to establish the key strategic elements necessary for a system based on artificial intelligence.

This study highlighted the existence of major contradictions related to the answers obtained, that may have been mainly due to bad decisions directly linked to the educational and professional training levels of the human resource. This human resource is in charge of ensuring the proper functioning and decision making of the supply chain, and two very important shortcomings were observed. One was the lack of higher technical education in the field related to Supply Chain 4.0, Logistics 4.0, and the fourth industrial revolution, and the other was regarding the great responsibilities that were given to these human resources lacking precisely the professional training according to the relevant needs. The fact of working in such a critical area as human health and not having the appropriate professional training is a cause for great concern due to the repercussions this may have on the patients treated there. With regard to the activities related to the logistics of healthcare centers, this is not only harmonized with support services such as delivery, warehouses, pharmacies or internal distribution, but also directly affects the services provided to patients on the ward, in consultation rooms, or in the surgical block. In fact, most of the activities that should be carried out by junior staff, such as orderlies, were assigned to clinical staff with a high degree of preparation or specialization in nursing, using direct action personnel on patients to perform operational activities, so that in practice, due attention was not paid to the professional activities for which these nurses were hired and the internal supply chain and patient care were often fragmented.

The inefficiencies that were detected within the logistics and hospital supply chain through the stock ruptures that these generated bacteraemia, are a concern at the institutional level and their elimination through the implementation of the use of tools directly related to the fourth industrial revolution, Supply Chain 4.0, Logistics 4.0, and AI would be key to the existence of a quality service and, at the same time, allow savings in operating costs, while releasing qualified personnel to fulfill the health functions for which they were hired. In addition, there is a need to maintain competent and updated systems, so that they complement the achievement of the goals of each health center.

It is important to emphasize relevant aspects of the research, such as the timely administrative and financial information provided by proper inventory control management. The flow of information, as well as the continuous application and improvement provided by AI for the improvement of process control, which has grown significantly in recent years. These are elements that provide opportunities for the development of more optimal solutions in processes where conventional control techniques are complex and subject to numerous errors.

There are definitely inefficiencies in management and decision making due to human failures during all the processes of the supply chain and logistics of hospitals in northern Chile. It is at this point where the incorporation of an AI is a potential solution, since it eliminates a large amount of human errors along with providing real-time information. The creation of an AI specialized in the supply chain, whose main parameters are based on the failures identified in this study, would help in decision making, the efficiency of the internal processes of the supply chain, and would avoid stock ruptures generated by human or management errors within the health centers.

Three of the four reasons most frequently selected by survey participants were due to internal shortcomings in logistics processes, from data errors, to product spoilage, to supplier non-compliance. All were negative concepts related to internal logistics problems and human error. This could be avoided even with a more exhaustive control of these errors, as well as of the workers, but this last option requires an extra investment (for payment of human resources for control, specific workshops, and extra measures, among others) that the centers were not willing to face. Therefore, investment and constant improvement of workers is required, but even so, there could still be errors since humans are prone to failure, despite the investment, in a natural way.

If compared with the different experiences in the literature, it can be observed that those industries that make use of AI, Logistics 4.0, and Supply Chain 4.0 showed considerable improvements in the efficiency of all the processes involved in the supply chain, in addition to making substantial savings in human resources expenses, loss of materials, misplacement, stock breakage, subtractions, excess of supplies in warehouses, among other benefits. In addition, they allowed immediate access to information at all levels of the organization, which facilitated decision making for all employees. This feature had a direct impact on planning, along with focusing control on a smaller number of key points, data entry, decision making, AI itself, and the execution of strategic planning. This reduced costs and response times to stock ruptures. All of the above meant an improvement in the delivery and quality of services to the end user and an internal improvement in the supply chain. This was due to the fact that a large part of its framework was automated and in constant improvement of the results delivered, given the nature itself, where data entry was the engine of improvement of the IAs. Therefore, its use in hospital centers is of utmost urgency in the short term, not only for Chile, but for the whole world.

At this point, when making the comparison of both states, we find efficiency improvements, substantial time reductions, among others, in the processes carried out in the supply chain, so that the incorporation of a specialized AI in the hospital supply chain will make improvements in the development of management and decision making within the health center. In addition, the use of AI decreases the dependence on human labor in these processes, perhaps not by one hundred percent, but by a considerably high percentage, thus avoiding errors or stock ruptures that are inherently generated when humans interact with the environment.

This leads us to think about future research that broadens the spectrum related to the use of AI for the management control of medical supply chains, their logistics, and health institutions in general, considering a broader sampling that allows replication on a nationwide and international scale.

## Data Availability

Data sharing not applicable.

## Appendix A

Chilean map and section of the northern region where the study was carried out [38].

This figure shows the political map of Chile in its north-south extension where the localities that were addressed in the investigation are highlighted with an orange rectangle.

## Appendix B. The Original Questionnaire Applied to the Experiment Was in Spanish

1. E-mail address.
2. Level of education.
3. Do you know the number of employees working in the Procurement sector? If you answered “Yes”, please specify the number.
4. How long have you been working for the health center?
5. With respect to the supply sector, does it use standards or procedures? If you answered “Yes”, please specify broadly what standards or procedures you are familiar with.
6. Are the organization’s supply control system methods modernized?
7. Do you know if the health center’s supply unit carries out an inventory in its warehouses? If you answered “Yes”, please specify the interval at which this is carried out.
8. Do you know what procedures are applied in the reception of products (drugs and supplies) for storage? If you answered “Yes”, please outline these procedures.
9. Do you know how the stored products are controlled? If you answered “Yes”, please outline such procedures.
10. Do you understand the procedures and routines of the supply unit activities? If you answered “Yes”, please outline these procedures.
11. Do you know what are the main activities involved in the replenishment of hospital supply stock? If you answered “YES”, please describe the phases involved in this procedure.
12. Are you aware of the standards recommended in the facility for the storage of products and materials? If your answer above was “YES”, do you consider that the storage space for products and materials complies with the recommended standards?
13. Do you understand the activities of the supply sector?
14. Do you understand how the supply chain information flow is distributed? If you answered “Yes”, please explain.
15. How is the distribution process of the health center’s supplies and drugs performed?
16. Does the health center promote staff training and/or education? If so, how many trainings does it have and in what area?
17. Regarding stock storage, what are the main tools or programs used for its organization?
18. Are there internal policies defined by the health center for supply management?
19. Are you familiar with inventory management techniques and if they are applied in the sector?
20. Is there regular monitoring of procedures directly related to the supply chain? If so, how often is this done?
21. How much do you know about artificial intelligence?
22. Do you believe that the inclusion of an AI system can help in daily tasks and resource management? Please justify your answer.
23. Of the following options that negatively affect inventory stock, check which one(s) you can currently identify in your organization.

**Absence of an adequate planning tool**
Data errors
Product deterioration
Limited warehouse space
Low availability of funds for inventory procurement
Inadequate ordering system
Lack of a purchasing management model
Non-compliance of suppliers
Randomness of demand
Randomness of delivery times
Insufficient production capacity to meet demand
